# Tuberculous Meningitis in Children and Adults: A 10-Year Retrospective Comparative Analysis

**DOI:** 10.1371/journal.pone.0133477

**Published:** 2015-07-17

**Authors:** Egidia G. Miftode, Olivia S. Dorneanu, Daniela A. Leca, Gabriela Juganariu, Andra Teodor, Mihnea Hurmuzache, Eduard V. Nastase, Dana T. Anton-Paduraru

**Affiliations:** 1 Department of Infectious Diseases, University of Medicine and Pharmacy “Grigore T. Popa”, Iasi, Romania; 2 Department of Microbiology, University of Medicine and Pharmacy “Grigore T. Popa”, Iasi, Romania; 3 Department of Pediatrics, University of Medicine and Pharmacy “Grigore T. Popa”, Iasi, Romania; University of Pittsburgh Center for Vaccine Research, UNITED STATES

## Abstract

**Background:**

Tuberculous meningitis (TBM) is the most lethal form of *Mycobacterium tuberculosis* infection, which has a high rate of neurological complications and sequelae.

**Objectives:**

Our study offers a real-world infectious disease clinic perspective, being thus representative for the clinical environment of developing countries.

**Methods:**

We performed a retrospective analysis of the 127 adult and 77 pediatric cases diagnosed with TBM in the Infectious Disease Hospital of the School of Medicine of Iasi, Romania between 2004–2013.

**Results:**

Definite diagnosis of TBM was established in 31% of children but in only 20% of adults (p = 0.043). A contact with an individual with pulmonary tuberculosis was documented in 30% of children *vs*. 13% of adults (p = 0.0007). Coma occurred in 19% of patients (similar in children and adults); other consciousness abnormalities were seen in 27% of children and in 72% of adults (p = 0.000001). Cranial nerve palsies occurred prior to therapy in 9% of cases (12% *vs* 7% of children and adults, respectively, p>0.05), and developed 2–7 days after treatment initiation in 10% (12 vs 9%). CSF cultures were positive for *M*. *tuberculosis* in 24% of patients (31% *vs*. 20%, p>0.05). Overall mortality was 7.35%, similar for children and adults. Yet, permanent neurological sequelae, which were seen in 23% of patients occurred significantly more frequent in children vs. adults (36% *vs*. 14%, respectively, p = 0.0121). In conclusion, our retrospective analysis on a significant number of cases of TBM identified striking differences between children and adults: while children were in an earlier stage at the admission, they associated a higher frequency of neurological sequelae and miliary pattern, and they were more likely to have normal CSF protein levels and positive cultures of CSF.

## Introduction

Tuberculosis has continued to spread unabated for too long. In 2011 there were an estimated 8.7 million new cases of tuberculosis (TB) worldwide, and 1.4 million people died from TB [1]. Central nervous system (CNS) tuberculosis accounts for approximately 1% of all cases of active tuberculosis and associates a high mortality and residual neurologic sequelae, even with adequate treatment [2, 3].

In Romania, the overall incidence of tuberculosis was approximately 100 cases per 100,000 population in 2011, with a decreasing trend from an incidence of 142 per 100,000 population in 2002, but the number of severe cases of TB in children (meningoencephalitis, miliary, cavitary) still maintains a high annual rate (65/100,000 in 2011) [4].

Natural history and clinical manifestations of tuberculosis are different in children and in adults [5], with a higher incidence of disseminated forms and a higher risk to progression to severe forms being reported to occur in children [5]. Note, however, that these differences are reported in different studies and that, in general, there are no unified studies carried out to directly compare the outcome of tuberculosis in the same setting in the same time and in the same diagnostic and treatment conditions.

In our region, different from other medical systems, where TB patients are hospitalized in separate wards (in pediatric hospitals and adult wards of infectious diseases or pneumophtisiology), children and adults are hospitalized in the same ward. This allows us to gain a global vision and compare and contrast the clinical and pathological features of TB meningitis in the two age categories to design appropriate diagnostic followed by an early introduction of treatment.

Here, we present our 10 year experience of managing adult and pediatric patients diagnosed with TBM in Northeast Romania. It is important to stress that, while in the recent years the diagnostic and interventional abilities dramatically improved in Romania, such modern and sophisticated diagnostic tools for TBM were not available in the past. Therefore, our study offers a real-world infectious disease clinic perspective, being thus representative for the clinical environment of developing countries. Such an approach might be useful not only to develop diagnostic and therapeutic guidelines applicable to poor resource settings, but also to identify those critical needs that have to be addressed to dramatically improve the diagnostic and treatment of patients with TBM in the developing world.

## Patients and Methods

### Ethic statement

The study’s objectives and procedures were approved by the Ethics Committee of the Hospital of Infectious Diseases (No. 1/January 2014), Iasi. No written consent was given by the participants because the data were analyzed anonymously.

### Study group

Our study retrospectively reviewed the case records of 204 HIV uninfected patients (77 children and 127 adults) consecutively hospitalized and treated for tuberculous meningitis (TBM) in the University Hospital of Infectious Diseases in Iasi–Romania during a ten-year period (2004–2013). Presenting clinical features, radiological abnormalities, cerebrospinal fluid (CSF) findings and the outcome of TBM was compared and contrasted between pediatric and adult patients.

Because the diagnosis criteria for TBM are not distinctly defined for adults and children in the medical literature, our major goal was to identify common features and differences that could help improve our ability to diagnose TBM very early and to prevent the development of neurological complications.

### Diagnostic criteria

Patients were stratified as having a definite, probable or possible diagnosis of TBM by using the consensus clinical case definition [6]. We used the scoring system proposed by Marais et al. [6] that includes *(i) clinical criteria (maximum 6 points)*: symptoms duration of more than 5 days (4p), systemic symptoms of tuberculosis (2p), history of tuberculosis/contact with a person with tuberculosis/positive tuberculosis skin test (TST) or positive interferon-gamma release assay (IGRA) (in children < 10 years) (2p), focal neurologic deficit (1p), cranial nerve palsy (1p) and altered consciousness (1p). *(ii) Cerebrospinal fluid (CSF) criteria (maximum 4 points)*: clear appearance, 10–500 cells/mmc, lymphocytic predominance, protein concentration ≥1 g/l and CSF to plasma glucose ratio <50%) (1 point, respectively); *(iii) Cerebral imaging criteria (maximum 6 points)*: hydrocephalus (1p), tuberculoma (2p), infarctus (1p), basal meningeal enhancement (2p), precontrast basal hyperdensity (2p). *(iv) Evidence of tuberculosis with other location (maximum 4 points)*: chest X ray suggestive of active tuberculosis (2p), miliary tuberculosis (4p), tuberculosis outside the CNS (2p), acid fast bacilli/*M*. *tuberculosis* positive from another source (4p).

While not included in this score, we also considered the results of the interferon-gamma release assay (IGRA) [QuantiFERON-TB Gold test (Cellestis Limited, Australia)]. This test was performed on a limited number of samples (44 adults and 15 children), as per manufecturer’s instructions.

According to this scoring system [6], a **definite diagnosis** of TBM was considered when patients presented with symptoms and signs of meningitis, and *M*. *tuberculosis* grew from CSF culture or a standard Ziehl-Neelsen stain revealed the presence of acid-fast bacilli (AFB).

**Probable TBM** was concluded for patients scoring ≥ 10 points (when cerebral imaging was not available) and ≥ 12 points (when cerebral imaging was available) with at least 2 points being contributed by CSF criteria.

**Possible TBM** was concluded for patients scoring 6–9 points without cerebral imaging and 6–11 points with cerebral imaging.

TBM staging was established using the method of Gordon and Parson [7], as follows: stage 1-fully conscious patients; stage 2- drowsy patients or presenting with focal neurological signs; and stage 3-comatose or nearly comatose patients.

### Statistical analysis

The chi-squared test was used for statistical analysis and a value of < 0.05 was considered statistically significant.

## Results

### Study groups

Over a 10-year period, we followed 204 patients with TBM, non-HIV coinfected. Seventy-seven patients were children and 127 were adults. Patient age at the presentation was in average 6.86±5.17 years (range: 3 months-15 years) for children. In this group, the median interquartile range (IQR) of age was 7 (1.79–11.5). Average age of the adult patients at the time of TBM diagnosis was 43±16.63 years (range: 18–87 years). In this group, the median IQR of age was 43 (29–55).

Sex distribution of pediatric cases was 61% (n = 47) male and 39% (n = 30) female. Similarly, in adult patients there was a slight, insignificant (p = 0.696) overrepresentation of male patients: 58% (n = 74) *vs*. 42% (n = 53) for male *vs*. female adult patients, respectively. When patients were stratified according to their origin, there was a clear higher risk for TBM in patients of rural origin. Thus, 159 (78%) cases were from rural areas, compared to only 45 (22%) from urban regions (p = 0.001). There were no significant difference between adults and children regarding their origin, with 88% of children and 71% of adult patients originating from rural regions.

Among potential risk factors associated with TBM, chronic diseases (liver and kidney diseases, neoplastic disease and immunosuppressive therapy) were more frequent in adults (25%) than in children (5%) (p = 0.0008). Conversely, a higher proportion of cases reporting contact with a person with tuberculosis could be identified in children compared to adults (30% *vs*. 13%, respectively, p = 0.0007).

### Clinical stage

The vast majority of patients (>62%) were in the stage 2 of TBM at admission. A smaller fraction of patients was in the first stage (15%). There was a significant difference in the stage of infection at the time of admission between children and adults. Thus, >25% of children and only 8% of adults (p = 0.00012) were diagnosed very early in the infection.

The observation that children with TBM are admitted in earlier stages of infection compared to adults may have direct clinical implications, as a late diagnosis or a late presentation to the hospital may dramatically affect future prognosis.

There were no significant differences with regard to the number of children and adults admitted in stage 3 (23.08% *vs*. 27.46%) (p>0.05) (Table 1).

**Table 1:** Epidemiological aspects, case definition and stage of TBM on admission.

	Children *(n = 77) n (%)*	Adults *(n = 127) n (%)*	Total *(n = 204) n (%)*	p value
**Case definition**				
Definite diagnosis	24 (31)	25 (20)	49 (24)	0.043
Probable TBM	26 (34)	35 (28)	61 (30)	> 0.05
Possible TBM	27 (35)	70 (55)	97 (48)	0.044
**Stage**				
Stage 1	20 (26)	10 (8)	30 (15)	0.00012
Stage 2	40 (52)	87 (68)	127 (62)	0.039
Stage 3	17 (22)	30 (24)	47 (23)	> 0.05
**Epidemiological aspects**				
Contact with TB	23 (30)	16 (13)	39 (19)	0.0007
History of TB	3(4)	32 (25)	35 (17)	0.0002
Poverty	15(19)	18(14)	33 (16)	> 0.05
Chronic disease	4(5)	32(25)	36 (18)	0.0008

Data are presented as n (%)

Definite diagnosis of TBM was more frequently established in children than in adults (31.16% *vs*. 17.32%) (p = 0.043), while criteria for a possible TBM diagnosis were predominant among adults (p = 0.044).

A comparison of the presenting clinical features and outcome in the 77 children and 127 adults with TBM is shown in Table 2.

**Table 2:** Clinical features and infection outcome in patients with TBM.

	Children (n = 77) n (%)	Adults (n = 127) n (%)	Total (n = 204) n (%)	P value
**Clinical findings**				
Average duration onset–admission (days)	9.1(4–27)	9.2 (3–32)		> 0.05
**Onset**				
- insidious	49 (64)	85 (67)	134 (66)	> 0.05
- sudden	29 (38)	55 (43)	84 (41)	> 0.05
Fever	56 (73)	104 (82)	160 (78)	> 0.05
Headache	46 (60)	98 (77)	144 (71)	> 0.05
Vomiting	39 (51)	41 (32)	80 (39)	0,042
Confusion	21 (27)	90 (71)	111 (54)	0.000001
Systemic symptoms	6 (8)	31 (25)	37 (19)	0.0053
Cough	10 (13)	18 (14)	28 (14)	> 0.05
**Coma**				
-on admission	14 (18)	22 (17)	36 (18)	> 0.05
- in evolution	4 (5)	13 (10)	17 (8)	> 0.05
Seizures	11 (14)	11 (9)	22 (11)	> 0.05
Cranial nerve palsy				
- on admission	9 (12)	8 (6)	17 (8)	> 0.05
- in evolution	9 (12)	12 (9)	21 (10)	> 0.05
**Outcome**				
Full recovery	43 (56)	100 (79)	143 (70)	0.051
Death	6 (8)	9 (7)	15 (7)	> 0.05
Neurological sequelae	28 (36)	18 (14)	46 (23)	0.0121
Drug-induced hepatitis	3 (4)	18 (14)	21(10)	0.0145

Data are presented as n (%)

The vast majority of included children received BCG prophylaxis prior to current infection. Only 19% of patients had systemic signs of bacillary disease (weight loss, night sweats, lethargy, etc.). There were relevant differences between children and adults regarding the systemic signs of bacillary disease (8% *vs*. 25%, for children and adults, respectively, p = 0.0053).

Neurological state at the time of admission was: coma in 36 patients (18% children *vs*. 17% adults, p>0.05); other consciousness abnormalities (dizziness, confusion, changes in personality) were observed more frequently in adults (n = 90, 71%) than in children (n = 21, 27%) (p = 0.000001). Cranial nerve palsies were noted prior to therapy initiation in 17 cases (12% *vs*. 6% for children and adults, respectively, (p>0.05), and developed after 2–7 days of treatment in additional 21 patients (10%) (12% *vs*. 9% children and adults, respectively).

The average duration of symptoms before hospitalization was 9 days (range: 3–32 days) and was not significantly different between children and adults (Table 2).

Extrameningeal TB presented most frequently as pulmonary tuberculosis, in 80 cases (39%) (35% *vs*. 42% of children and adults respectively, p = 0.461), as well as adenitis in 3 cases and spondilodiskitis in 5 adults cases.

Major findings on chest radiological exam (Table 3) consisted of miliary pattern, observed in 32 patients (16%) (20% *vs*. 13%, of children and adults, respectively, p = 0.144), fibrocavitary lesions, diagnosed in 19 (9%) patients (3% children *vs*. 13% adults, p = 0.0174), upper lobe infiltration, in 16 patients (8%) (4% children *vs*. 10% adults) (p>0.05) and pleural effusions in 8 cases (4%) (3% children *vs*. 5% adults, p = 0.528) (Table 3).

**Table 3:** CSF data and chest X-ray abnormalities.

	Children (n = 77) n (%)	Adults (n = 127) n (%)	Total (n = 204) n (%)	p value
**Neutrophils>50%**	3 (4)	14 (11)	17 (8)	0.016
**Protein (g/l) (normal value < 0.4 g/l)**				
0.4–1	24 (31)	18 (14)	42 (21)	0.0095
1.1–2	27 (35)	39 (31)	66 (32)	> 0.05
2.1–3	13 (17)	22 (17)	35 (17)	> 0.05
3.1–5	11 (14)	25 (20)	36 (18)	> 0.05
> 5	2 (4)	23 (18)	25 (12)	0.0021
**Glucose (g/l) (normal value ≥ 0.4 g/l)**				
**≥** 0.4	22 (29)	47 (37)	69 (34)	> 0.05
0.3–0.4	24 (31)	34 (27)	58 (30)	> 0.05
0.2–0.3	31 (40)	46 (36)	77 (41)	> 0.05
**Chloride (normal value 6.8–7.2 g/l)**				
5–5.6	4 (5)	11 (9)	15 (8)	> 0.05
5.6–6.7	38 (49)	59 (47)	97 (48)	> 0.05
6.8–7.2	35 (46)	57 (45)	92 (45)	> 0.05
*M*. *tuberculosis* isolation	24 (31)	25 (20)	49 (24)	> 0.05
**Chest X-ray**				
Abnormal aspects	27 (35)	53 (42)	80 (39)	> 0.05
Miliary	15 (20)	17 (13)	32 (16)	> 0.05
Fibrocavitary	2 (3)	17 (13)	19 (9)	0.0174
Infiltrative	3 (4)	13 (10)	16 (8)	> 0.05
Pleural effusion	2 (3)	6 (5)	8 (4)	> 0.05
Hilar lymphadenopathy	5 (7)	-	5 (3)	

Data are presented as n (%)

Tuberculosis skin test elicited a positive reaction in 21 of 58 tested patients (36%)(35% *vs*. 37% of children and adults respectively, p = 0.889). CSF smear for acid-fast bacilli was negative in all the cases. CSF culture was positive for *M*. *tuberculosis* in 49 cases (31% of children *vs*. 20% of adults, p = 0.024), and a positive interferon-gamma release test [QuantiFERON-TB Gold test (Cellestis Limited, Australia)] was observed in 40 of serum samples out of 59 tested (60% *vs*. 68.2% for children *vs*. adult patients) (p> 0.05).

Upon admission, CSF, which was available in all cases of TBM, revealed turbidity in 20 patients (10%) (9% vs. 10% for pediatric *versus* adult patients, respectively) (p> 0.05). In the CSF, polymorphonuclear cells represented >50% of the total leucocyte population in 17 cases (4% *vs*. 11% for pediatric *vs*. adult patients, respectively) (p = 0.016). Glucose levels were normal (**≥** 0.4g/l) in the CSF at presentation in 69 patients (34%) (29% *vs*. 37% for pediatric *vs*. adult patients, respectively, p> 0.05). The initial CSF chloride levels were normal in 92 patients (46% *vs*.45% for pediatric *vs*. adult patients, respectively, p >0.05), with decreasing values during the first month of treatment in half of them. The protein CSF levels were elevated in all cases, ranging from 0.4 to 17 g/l (normal range 0.2–0.4 g/l), with very high levels (5–15 g/l) being more frequently seen in adults (18%) than in children (3%) (p = 0.0021).

Treatment was empirically instituted within a few hours to 3 days postadmission in the majority of patients and included a combination of four antituberculous drugs (isoniazid, rifampicin, ethambutol and pyrazinamide) during the initial 2–3 months and two drugs (isoniazid, rifampicin, 3 days weekly) for the next 7–9 months [8].

Drug related hepatic toxicity was recorded in 21 cases (10%) and was more frequent in adults than in pediatric patients (4% *vs*. 14% of children and adults respectively, p = 0.0145).

Isolates of *M*. *tuberculosis* were tested for isoniazid susceptibility only in 4 cases (one strain was resistant).

The overall in-hospital mortality for our group of patients was 7% (15 cases) (8% of children *vs*. 7% of adults, p>0.05) whilst permanent neurological sequelae (hydrocephalus, cranial nerve palsies, hemiplegia) were seen in 46 patients (23%), more frequently in children than in adults (36% *vs*. 14% for pediatric *vs*. adult patients, respectively, p = 0.0121). Patients were discharged after an average duration of hospitalization of 28 days.

## Discussion

TBM is the most lethal clinical presentation of *Mycobacterium tuberculosis* infection, with a mortality rate that may be exceptionally high (6–65%, average 33%), despite treatment with antituberculosis chemotherapy [9]. TBM is also responsible for severe disability in a vast proportion of survivors [9, 10].

In this study, we compared and contrasted the elements used for the diagnosis of TBM in children and adults with the same demographic conditions, the same limitations regarding imaging technology and exposed to the same strains of *M*. *tuberculosis*. The vast majority of these patients were admitted in critical condition in the Infections Disease Clinics of the University Hospital of Iasi, Romania. By using standardized clinical criteria we were able to early diagnose patients with TBM in the majority of cases, and to initiate specific treatment within the first days after admission. At the time of the diagnosis in these patients, resources in our hospital being limited, our group is illustrative for real-life practice in poor resources settings.

At first consultation, most of the patients in our study group presented in an already advanced stage of disease (stages 2 and 3), while only 14% of patients presented in stage 1, similar to previous reports by other groups [11, 12], but different from the data reported by Maltezou on a small sample size [13].

The explanation of this situation could be the low level of education and limited accessibility to specialized services (most of patients came from rural areas). Other reason for the delayed presentation may rely on misinterpretation by general practitioner and pediatricians of nonspecific signs and symptoms during the first week of disease (the involvement of CNS was suggested later, when meningeal signs occurred). Moreover, especially in children, late presentation might have been due at least partially to a characteristic rapid progression to severe forms [5].

In our ten-year survey, 24% of patients were found to have positive cerebrospinal fluid cultures for *M*. *tuberculosis*, with a higher rate in children than in adults (p>0.05), in agreement with previous studies [14, 15, 16]. Thus, Thwaites et al., reported a 29% positivity rate for *M*. *tuberculosis* cultures in 432 uninfected HIV patients with TBM [14] and in a recent study, Blount et al., noted a culture confirmation rate of 27% in children with intra and extrathoracic tuberculosis [17]; Rajeev and Desai found similar positivity rates in India [15, 16]. Conversely, Gijs et al., reported only 11.7% positive *M*. *tuberculosis* cultures in South African children [12].

Multiple studies reported that miliary tuberculosis is directly involved in the pathogenesis of tuberculous meningitis in children. In our study nearly 20% of pediatric patients had evidence of miliary tuberculosis, strongly suggesting CNS involvement [10]. These rates were significantly higher than those recently reported by Hristea et al., on patients from Southern Romania, in which miliary tuberculosis was associated to only 7% of cases of MTB [18].

In our study, the most consistent difference regarding the chest X-ray abnormality between the two groups of patients were the fibrocavitary aspect, more frequently seen in adult (13%) than in pediatric patients (3%)(p = 0.0174).

Definite diagnosis of TBM was more frequently achieved in children (31%) than in adults (17%). This difference may rely on the frequent miliary pattern and the highest rate of successful *ex vivo* isolation of *M*. *tuberculosis* in children, which were similar to a previous report of Thwaites et al., in an uninfected HIV population with TBM in Vietnam [13].

Unlike the results of other investigators [10, 12, 19], in our study the proportion of children presenting with coma, seizures and cranial nerve palsies was similar to that observed in adults (18% *vs*. 17%, 14% *vs*. 9% and 12% *vs*. 6%, respectively). MTB also frequently (80%) associated an abnormal mental state, but in children, coma and seizures were noted in a much lower proportion than in the recent study of Blount [17]. The duration of symptoms before admission was relatively short (9 days). Both these clinical aspects were different from those previously reported by Hosoğlu et al. [19].

Biomarker testing also revealed several remarkable features of MTB infection in children and adults: (i) no relevant difference was found between children and adults regarding CSF biomarkers, except for higher CSF protein content in adults (> 5 g/l); (ii) similarly, the levels of chloride and glucose in the normal range were similar between children and adults; this pattern could explain the delay of treatment in TBM. These observations might have a major therapeutic impact as, in the absence of significant changes of the CSF biomarkers, the decision to initiate treatment might be delayed, which may be reflected in a poor survival rate.

MTB complications were significantly more likely to develop in children (36%) and hydrocephalus was the most frequent of them (26%). This is a common complication of TBM which was reported to occur in 15–18% of adults and 57–98% of children [10, 11, 20, 21].

IFN-γ release assay was positive in 68% of cases and had a higher specificity than tuberculin skin test. Yet one of the limitations of the IFN-γ release assay is that it cannot distinguish between *M*. *tuberculosis* infection and active disease [6].

Decision to start antituberculosis treatment was empirical in all cases. WHO guidelines recommend a 6 or 9 months course of antituberculosis treatment [1]; however, other guidelines recommend a prolonged therapy extended to 9 or 12 months if low suspicion of drug resistance or up to 24 months if high suspicion of drug resistance. Association of corticosteroids may reduce the number of deaths [8, 22]; therefore, all the patients in our cohort received corticosteroids associated to specific antituberculosis treatment. Similar with the data from a recent systematic review by Donald [23] we found that drug-induced hepatotoxicity was considerable lower in children than in adults. In the same review, when considered papers describing antituberculous drug hepatotoxicity, authors noted variable frequency from less than 1% to 50% in some studies involving children treated for tuberculous meningitis, differences explained by disease severity [23].

Clinical deterioration after administration of antituberculosis drugs is a common feature in TBM, and it is known as “paradoxical”. In our study, cranial nerve palsy occurred in 21 (10%) and coma developed in 17 (8.33%) after the introduction of adequate therapy, without relevant differences between children and adults. Note that these features might be due to bactericidal drugs, such as isoniazid, which may cause the diffuse release of bacillary contents which is responsible of inflammation and edema [10, 24]. Despite a dramatic initial course, 70% of our patients responded promptly to the treatment with antituberculosis drugs and subsequently fully recovered.

The in-hospital mortality rate was 7%, which is among the lowest rates mentioned in the literature (which range from 7 to 57%) [11, 13, 21, 25, 26]. The low mortality rate in our study could be an argument against a high level of resistance in North Eastern Romania. According to WHO recent report, levels of multidrug-resistant tuberculosis (MDR-TB) remain worryingly high in some counties, notably countries in Eastern Europe and Central Asia, where 9–32% of new cases have MDR-TB and more than 50% of previously treated cases have MDR-TB [1]. Another reason for the low mortality in our study may relay to the use of a rigorous, standardized diagnostic strategy in these patients. As such, our results demonstrate that even in a poor resource environment, prognostic of TBM may be acceptable if rigorous diagnostic strategies are in place.

**In conclusion**, adults were more frequently admitted in the late stages of the diseases and atypical CSF findings such as normal CSF chloride and glucose level were more common than in children. Conversely, children with TBM were more likely to be contacts of tuberculosis cases, to have a normal CSF protein level, a positive CSF culture, and an evolution with neurological complications in a higher proportion comparing to adults.

The rapid evolution towards a negative outcome of the TBM in our cohort strongly suggest that, in areas with a high incidence of TB an improved therapeutic success can be achieved by improving the diagnostic abilities by implementing rapid diagnostic tests such as interferon-gamma release test or PCR for *Mycobacterium* detection, as well as cerebral imaging. Altogether, these approaches may lead to an earlier initiation of treatment in patients with associated risk factors. This is particularly important for a clinical condition as TBM, which evolve quite a while before admission.
